# A Plant-Based Dietary Supplement Exhibits Significant Effects on Markers of Oxidative Stress, Inflammation, and Immune Response in Subjects Recovering from Respiratory Viral Infection: A Randomized, Double-Blind Clinical Study Using Vitamin C as a Positive Control

**DOI:** 10.3390/ijms26115209

**Published:** 2025-05-29

**Authors:** Bruno Fink, John M. Hunter, Zbigniew Pietrzkowski, Richard Fink, Coy Brunssen, Henning Morawietz, Boris Nemzer

**Affiliations:** 1 Noxygen Science Transfer & Diagnostics GmbH, 79215 Elzach, Germany; bruno.fink@noxygen.de (B.F.); richard.fink@noxygen.de (R.F.); 2 VDF FutureCeuticals, Inc., Momence, IL 60954, USA; jhunter@futureceuticals.com; 3 VDF FutureCeuticals, Inc. Irvine, CA 92606, USA; zb@futureceuticals.com; 4 Division of Vascular Endothelium and Microcirculation, Department of Medicine III, Medical Faculty Carl Gustav Carus and University Hospital Carl Gustav Carus Dresden, TUD University of Technology Dresden, 01307 Dresden, Germany; coy.brunssen@ukdd.de (C.B.); henning.morawietz@ukdd.de (H.M.)

**Keywords:** COVID-19, viral insult, cellular metabolic activity (CMA), ROS, endothelial dysfunction, circulating NOHb, inducible nitric oxide synthase (iNOS), uncoupled iNOS, phagocytic NADPH oxidase, FMD, dietary supplement PB-blend, influenza, seasonal virus, flu season

## Abstract

Respiratory viruses continue to present serious health challenges to human wellness. Growing evidence suggests that the more severe and damaging effects and symptoms of influenza, rhinovirus (RV), respiratory syncytial virus (RSV), and COVID-19 may primarily result from their common ability to disorganize the body’s healthy immune response. The simultaneous over-stimulation of several reactive oxygen species (ROS) pathways and concurrent suppression of bioavailable Nitic Oxide (NO) contribute to an immune disbalance that can lead to cellular oxidative distress and an excessive inflammatory response. This study evaluated the real-time, acute ability of a single, orally administered 50 mg encapsulated dose of a plant-based dietary supplement (“PB-Blend”), compared to 1000 mg of Vitamin C as a positive control, to modulate multiple ROS associated with a dampened immune response, as well as NO and other markers of inflammation, in a cohort recovering from a moderate course of COVID-19. This randomized, double-blind study was performed on 28 individuals 18–24 days after a moderate COVID-19 infection. Participants were orally supplemented with a single encapsulated dose of either 50 mg of PB-Blend or 1000 mg Vitamin C as a positive control. Changes in the levels of bioavailable NO (measured as circulating NOHb) were assessed, as well as the ex vivo cellular formation of mitochondrial, NOX2-, iNOS-, and TNFα-dependent ROS. All parameters were measured in real time before ingestion (baseline), and then at 30, 60, 120, and 180 min after administration. ROS were measured using a portable electron paramagnetic resonance (EPR) spectrometer. Inflammatory, immunity (hsCRP and TNFα plasma levels), interleukin (IL1, IL6, IL8, and IL10), cytokine (IFNγ, TNFα, and NF-κB), and immunoglobulin (IgA, IgM, IgG, and IgE) profiles were also followed. In addition to laboratory and cell function investigations, we performed clinical cardio ergometry, blood O_2_ saturation, and respirometry examinations. As hypothesized, the collected baseline data from this study group confirmed that mitochondrial, NOX2, and iNOS enzymatic systems were strongly involved in the generation of ROS at 18–24 days following a positive COVID-19 PCR test. Acute single-dose supplementation of 50 mg PB-Blend had a multifunctional impact on ROS and significantly inhibited the following: (a.) mitochondrial ROS levels by up to 56%; (b.) iNOS by up to 60%; and (c.) NOX2-dependent ROS generation by up to 49%. Moreover, 1000 mg Vitamin C supplementation exhibited narrower ROS-mitigating activity by solely inhibiting NOX2-dependent ROS generation by 45%. Circulating NOHb levels were significantly increased after PB-Blend administration (33%), but not after Vitamin C administration. PB-Blend and Vitamin C exhibited similar potential to reduce ex vivo high dose TNFα (200 ng/mL)-induced H_2_O_2_ formation. These results suggest that 50 mg of PB-Blend has the potential to modulate disbalanced mitochondria, iNOS, and NOX2 enzymatic systems that can be engendered during respiratory viral infection and subsequent recovery. Moreover, PB-Blend, but not Vitamin C, showed potential to upregulate bioavailable NO, which is known to decline under these conditions. Based upon these observations, PB-Blend could be considered an alternative to, or to be used in tandem with Vitamin C in applications that promote immune support and recovery during seasons of heightened respiratory viral risk (e.g., “flu season”).

## 1. Introduction

Every year, especially during the fall and winter months, humans are subjected to an onslaught of influenza, rhinovirus, and, more recently, respiratory syncytial virus (RSV) and COVID viruses. Collectively referred to as “seasonal viruses”, they share a number of common traits, and can induce similar pathologies [1,2,3,4,5,6,7], including the following: (a.) acute, significant increases (hyperstimulation) in multiple reactive oxygen species (ROS) pathways (i.e., an excessive immune response often referred to as “oxidative burst”) that can subsequently lead to oxidative distress [1], and (b.) the concomitant suppression of bioavailable nitric oxide (NOHb). In combination, these two conditions create an immune disbalance that can be accompanied by the release of excessive inflammatory cytokines and chemokines. The frequency and severity of these viruses seems to be linked to an unhealthy disruption of the body’s immune responses [8]. For example, it has been reported that over-stimulation by influenza virus can disrupt the intracellular redox balance and lead to the deterioration of cellular defenses [9]. Along similar lines, a direct correlation was reported between the severity of symptoms associated with rhinovirus (RV) infection, concentrations of interleukin-8 (IL8), and levels of NF-κB activation in nasal secretions, indicating that RV stimulation of IL8 in the respiratory epithelium may be mediated through the production of oxidative species and the subsequent activation of NF-κB [10]. At the terminal extreme, mortality from influenza is associated with serious complications caused by cytokine storms, such as acute respiratory distress syndrome (ARDS). Like COVID-19, influenza can also induce inflammation-promoting endothelial dysfunction, which can further exacerbate the cytokine storm [11]. It has also been observed that COVID-19 and influenza disease courses range from asymptomatic or mild [12] to more severe forms [13] with pulmonary and endothelial inflammation and thromboembolic complications, or to acute respiratory distress syndrome (ARDS) with or without multiorgan failure [14,15]. Other similarities between COVID-19 and influenza have been described [16]. Like influenza, over-abundant neutrophils in the lungs during RSV infection can undergo oxidative burst, thereby producing large amounts of ROS that can oxidize biomolecules, damage the epithelial–endothelial barrier and other host cellular structures, and promote lung injury [17]. Published manuscripts describe COVID-19 as an infection associated with the excessive production of cytokines and chemokines [18], activation of inflammatory cells, apoptosis of lung epithelial and endothelial cells, and other pathophysiological processes linked to cellular redox imbalances and excessive reactive oxygen species production [19,20,21]. There is significant cause, therefore, to consider these redox and immune disbalances as shared, underlying mechanisms common to all of these respiratory viruses. When viewed as a more comprehensive group, it can be seen that viral respiratory infections cause multiple complications to the respiratory, cardiovascular, renal, nervous, and immune systems [22] due to acute, often uncontrolled increases in numerous ROS associated with excessive immune responses [18]. These redox processes have essential implications in cell biology and can be engaged in reversible and irreversible changes in physiological redox regulation or oxidative distress since they participate in many bioenergetic, metabolic, and life functions [23]. Of particular importance in the progression of inflammation brought about by viral insult, however, is the activation of phagocytic NADPH oxidase (NOX2) and the expression of inducible nitric oxide synthase (iNOS) from inflammatory cells. These emerge as key regulators of host immune responses [24]. Consequently, modalities that could downregulate NOX2 and iNOS during the course of viral infection and recovery could be of interest and could influence symptomatic pathology. Due to the multiple enzymatic pathways known to contribute to redox disbalance, it was therefore plausible to postulate that a well-designed plant-based blend with established antioxidant activity might be beneficial during periods of active viral respiratory infection, and also during subsequent recovery, in order to help the body limit the undesirable over-expression of a broad spectrum of disrupted immune responses and their associated symptomatic progressions [9,25]. During our previous research, a proprietary, standardized, and industrially produced plant-based dietary supplement blend containing 29 fruit- and vegetable-based ingredients was previously shown to exert a wide range of antioxidant (scavenging) capabilities on multiple ROS, such as superoxide anion (O_2_^•−)^, hydrogen peroxide (H_2_O_2_), and hydroxyl radical (^•^OH) [26]. In another earlier clinical study, that same 29-ingredient blend was reported to exert significant reductive effects on real-time measures of ROS while simultaneously increasing the bioavailable nitric oxide (NOHb) [27]. In a subsequent study, a similar proprietary blend that had been reduced from the previous 29 ingredients to only seven of the most active fruit- and vegetable-based extracts and whole powders (the current “PB-Blend”) was reported to exert similar results [28]. Another study was subsequently conducted on randomized overweight and obese subjects. Based upon PB-Blend’s observed ability to reduce the baseline ROS levels associated with metabolic syndrome, and to increase the baseline levels of NOHb in overweight individuals during the 90 days of that study, we concluded that our next study model should include a population exhibiting more reliably elevated ROS baseline levels, generated by other conditions, while simultaneously examining other markers of interest. Such a study could provide deeper and more specific insights into PB-Blend’s activities [29]. This conclusion prompted us to consider a study population that had been exposed to respiratory viral insult. Consequently, we recruited a population recovering from a moderate course of COVID-19 infection. It was our premise that COVID-19 could serve as a reasonable proxy for the other seasonal respiratory viruses, since all of the above-named viruses similarly affect many of the pathways, parameters, and enzymes we examined here [1]. We hypothesized, based upon emerging reports, that a recovering COVID-19 group would be experiencing excessive ROS-based immune responses, similar to those experienced during typical seasonal viral insult. We also hypothesized that this population would exhibit reduced NOHb, increased NOX2 and, most critically from a disease progression vantage, increased iNOS-generated ROS. If so, our model could provide opportunities to evaluate any effects that the PB-Blend might have on the enzymatic systems involved in the virus-induced elevation in ROS that can cause metabolic dysfunction and reduction–oxidation reaction (redox) imbalances. Critically, these very imbalances may influence the severity, and in some cases even the occurrence, of symptoms so often experienced during viral infection.

## 2. Results and Discussion

*Baseline clinical and laboratory parameters of study subjects*: During the intake assessment of the clinical and laboratory parameters of this study, we observed normal systolic and diastolic blood pressure, heart rate, and partial oxygen pressure (see Table 1). Furthermore, we observed moderately elevated fasting glucose levels and LDL-cholesterol levels, a trend towards very slightly decreased flow-mediated vasodilatation (FMD), and a slightly decreased forced vital capacity (FVC). Interestingly, more than 85% of these volunteers subjectively complained of fatigue and a dramatic loss of strength. The study was performed during the third COVID-19 wave in Germany, when Alpha was the COVID-19 Variant of Concern [30]. Furthermore, positive COVID-19 PCR tests confirmed elevated average levels in an unvaccinated study population of 275 and 439 BAU/mL (antibody binding capacity per milliliter) Cov-2-IgG values. Concurrently, the average Cov-2-IgM values indicated a mild-to-moderate COVID-19 disease course (see Table 2).

**Figure 1:**
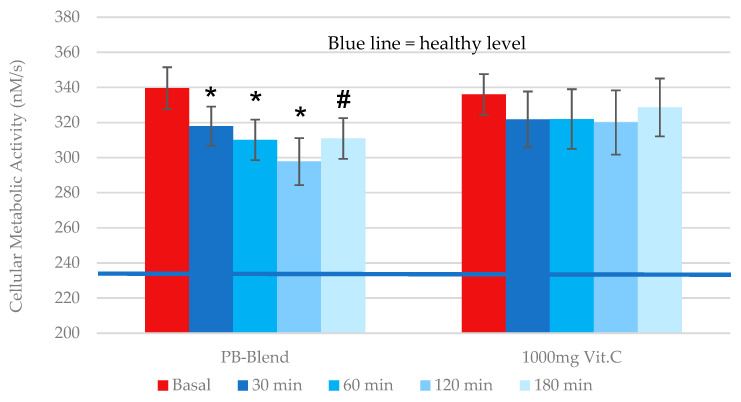
Time-dependent CMA values after oral single-dose administration of 50 mg of PB-Blend or 1000 mg of Vitamin C in subjects recovering from COVID-19 infection. CMA (generation of ROS) was determined in human whole-blood samples using NOXYSCAN EPR system, and partially in parallel with the portable VitaScreen electron spin resonance analyzer, 18–24 days after the diagnosis of COVID-19. The CMA values were highly elevated in the PB-Blend group (339.6 +/− 11.9 nM/s) and in the Vit. C group (336.0 +/− 11.6 nM/s) compared to values typically observed in healthy persons (as represented by the blue line) (CMA < 235 nM/s [29,32]). Data are shown as mean +/− standard error of the mean (SEM, *n* = 14 per group). Symbols *—*p* < 0.01 or #—*p* < 0.05, indicating statistical significance vs. baseline value prior to supplementation.

*Total cellular metabolic activity*: Our data show that volunteers had extremely high baseline CMA levels of up to 340 nM/s at intake (see Figure 1). These CMA levels strongly suggest the vigorous activation of inflammatory defenses, the generation of ROS through leucocytes, monocytes, and neutrophils possessing phagocytic NADPH oxidase (NOX2), and mitochondrial dysfunction as a consequence of excessive distress [31]. These conditions are highly energy consuming and could possibly account for the feelings of fatigue and weakness reported by the volunteers at intake.

After the single-dose supplementation of 50 mg of PB-Blend we observed the significant, time-dependent inhibition of ROS generation with maximal efficacy after 2 h of ingestion. Similar pharmacodynamics were previously described for PB-Blend in overweight and slightly obese individuals. In that study, the mitochondrial-dependent generation of ROS was revealed to be the major dysfunctional pathway [29].

Comparatively, the administration of a single dose of 1000 mg of Vitamin C resulted in a non-significant trend towards decreasing CMA. 

**Figure 2:**
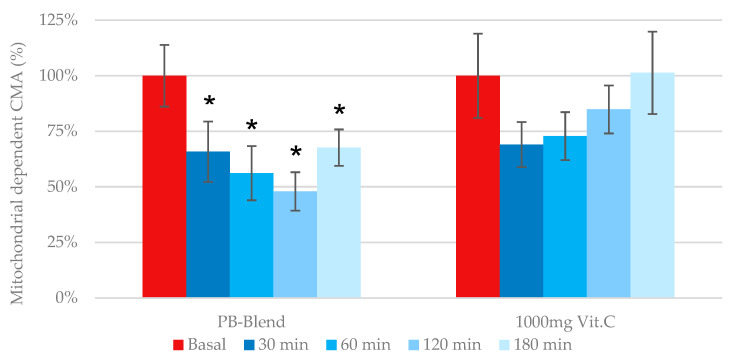
Time-dependent effects of a single dose of 50 mg of PB-Blend or 1000 mg of Vitamin C on eCMA-MITO values of subjects with a moderate course of COVID-19 infection. Results are presented as percent changes (deltas) from baseline. The eCMA-MITO was determined as the portion of the same human whole-blood sample after adding Antimycin A (10 µM), a selective complex III mitochondria respiration chain inhibitor. Study volunteers received a single oral dose of PB-Blend or Vitamin C 18–24 days after diagnosis of COVID-19. Data are shown as mean +/− standard error of the mean (SEM, *n* = 14 per group). Symbols *—*p* < 0.01, indicating statistical significance vs. baseline value prior to supplementation.

*Mitochondrial-dependent cellular metabolic activity*: Previous work has identified that mitochondria-dependent ROS generation becomes elevated during several pathologies, including metabolic syndrome, cardiotoxicity, and again particularly relevant to this investigation, a disbalanced immune response due to viral infection (see second Figure in Section 3.2). As previously illustrated, the measurement of eCMA-MITO is critical during the assessment of any exogenous antioxidant’s potential to provide immune support. According to our data, the single-dose administration of PB-Blend significantly improved mitochondrial function after 30 min of administration and reached maximal effect after 2 h (see Figure 2). Comparatively, e-CMA-MITO did not reach significance following the administration of a single 1000 mg dose of Vitamin C.

*iNOS-dependent cellular metabolic activity*: The bioavailability of the ubiquitous signaling radical, nitric oxide (NO), is regulated by the activity of three different NO synthases that influence critical functions: endothelial (eNOS), neuronal (nNOS), and the inducible (inflammatory) NOS (iNOS). In contrast to eNOS and nNOS, inducible NOS can produce a 20-times greater amount of NO and can play a key role in fighting viral infection [33,34]. Many cells are capable of expressing iNOS, including fibroblasts, hepatocytes, endothelial, and epithelial cells [35]. iNOS expression can be induced by several agents, including microbial lipopolysaccharides and virally induced cytokines, including RSV, influenza, and COVID-19.

In healthy people, iNOS-elevated NO levels provide an appropriate, targeted response to viral and bacterial incursion, environmental challenges, and other pathologies, and their action is generally limited to the viral body. Significant increases in baseline ROS levels due to chronic systemic insult, and especially if exacerbated by age- or lifestyle-related comorbidities such as obesity, metabolic imbalances, diabetes, cardiovascular diseases, smoking, or alcohol abuse can, over time, elicit unfocused, un-coordinated, and excessive inflammatory iNOS responses. However, when aggravated by acute viral insult, disruptive iNOS excesses can then further lead to a pronounced cascade of NO generation. In the face of highly elevated and sustained iNOS, the subsequent excessive release of untargeted NO results in rapid conversion of NO into superoxide (O_2_^−^) and peroxynitrite (ONOO^−^) [k = 1.6 × 10^9^ M^−1^s^−1^] [36,37]. This further limits the available pool of beneficial NO while simultaneously exacerbating the pro-oxidative redox status of the individual. Particularly during COVID-19, this has been shown to trigger the excessive formation of cytokines [18].

Consequently, we used the eCMA-iNOS assessment in order to determine (a.) whether our study group, after a moderate COVID-19 course, exhibited elevated expressions of iNOS at baseline and (b.) whether PB-Blend or Vitamin C had any effect on iNOS levels post treatment. As hypothesized, the baseline data from both groups of volunteers showed elevated eCMA-iNOS levels, which would be atypical in healthy individuals. Our data confirm that iNOS-driven ROS are a key factor in viral insult pathology. Additionally, as demonstrated in this study, the measurement of iNOS-driven ROS could be utilized as a useful marker during the medical evaluation of the relative severity of viral infection. Our data confirmed that (a.) iNOS was expressed during a moderate COVID-19 course and (b.) the expression of iNOS could lead to the generation of ROS.

**Figure 3:**
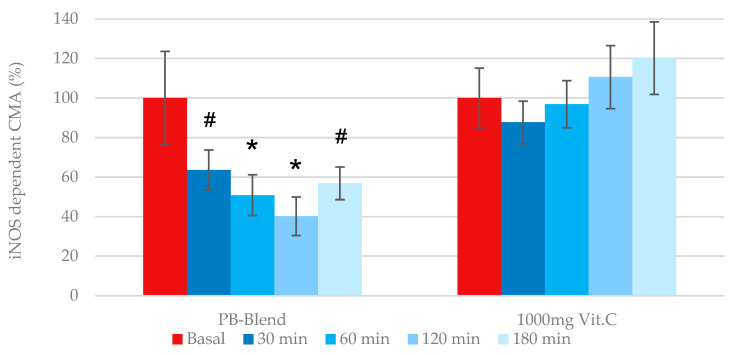
Single dose effects of 50 mg of PB-Blend or 1000 mg of Vitamin C on time-dependent eCMA-iNOS values of subjects having a moderate course of COVID-19 infection. The eCMA-iNOS was determined in the portion of the same human whole-blood sample after adding 1400 W (100 nM), a selective inhibitor of inducible nitric oxide synthase (iNOS). Data here represent the generation of ROS by dysfunctional or “uncoupled” iNOS, which is a consequence of excessive, cytokine-induced oxidative distress. Study volunteers received a single dose of PB-Blend or Vitamin C 18–24 days after diagnosis of COVID-19. Data are shown as mean +/− standard error of the mean (SEM, *n* = 14 per group). Symbols *—*p* < 0.01 or #—*p* < 0.05, indicating statistical significance vs. baseline value prior to supplementation.

The oral administration of 50 mg of PB-Blend, similar to its previous inhibition of eCMA-MITO, demonstrated a time-dependent inhibition of iNOS-derived ROS with a maximal observed effect after 2 h (Figure 3). Further observation after 2 h showed a gradual attenuation of iNOS inhibition. Comparatively, the oral administration of 1000 mg of Vitamin C did not reduce iNOS-dependent ROS generation, and in fact, tended towards propagating it during later time points.

**Figure 4:**
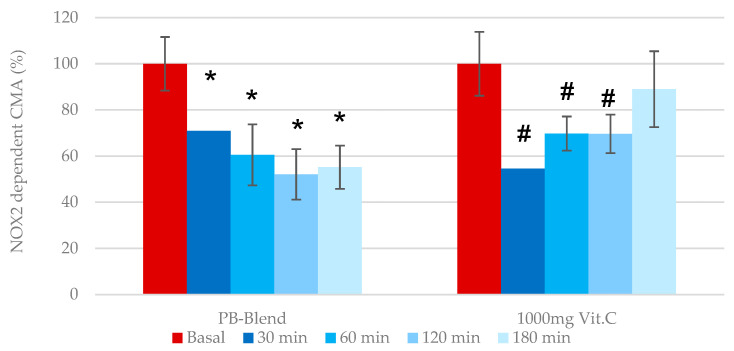
Time-dependent effects of a single dose of 50 mg of PB-Blend or 1000 mg of Vitamin C on phagocytic NADPH oxidase NOX2-CMA values of subjects after 18–24 days of a moderate course of COVID-19 infection. The NOX2-CMA was determined in a portion of the same human whole-blood sample after adding 10 µM Apocynin, a selective inhibitor of NOX2. NOX2-CMA values here represent the activation of NOX2 within inflammatory cells induced by COVID-19 infection. Data are shown as mean +/− standard error of the mean (SEM, *n* = 14 per group). Symbols *—*p* < 0.01 or #—*p* < 0.05, indicating statistical significance vs. baseline value prior to supplementation.

*NOX2-dependent cellular metabolic activity*: Under normal conditions, NOX2-derived O_2_^−^ regulates many aspects of innate and adaptive immunity, including the regulation of type I interferons, phagocytosis, antigen processing and presentation, and cell signaling. Phagocytic nicotinamide adenine dinucleotide phosphate (NADPH) oxidase (NOX2) is an enzyme that generates superoxide from molecular oxygen, utilizing NADPH as an electron donor [38]. Several studies have demonstrated that increased neutrophil-to-lymphocyte ratios, accompanied by an elevation in O_2_^−^ generation, correlate with more severe disease outcomes [39]. During pathological conditions such as SARS-CoV-2 and other respiratory viral infections, activated neutrophils have been shown to be one of the main sources of ROS production [40]. Consequently, specific inhibitors of NOX enzymes have become of interest as possible therapeutics. Numerous pan-NOX inhibitors have been developed in the past, whereas others are specific to one NOX enzymes [41]. In order to determine whether the PB-Blend may act to inhibit NOX2, we employed the eCMA-PHAGO-NOX2 assay. Apocynin (10 µM), a selective NADPH oxidase inhibitor, was added to collected whole-blood samples. The single-dose administration of 50 mg of PB-Blend demonstrated a nearly 40% decrease in O_2_^−^ generation after 2 h, and continued through 3 h (Figure 4).

In comparison, 1000 mg of Vitamin C reached a maximal efficacy of up to 36% after 30 min of administration, but diminished after approximately 3 h, leading to a loss of antioxidative capacity. Vitamin C’s scavenging capacity of superoxide (pK—6.3 10^3^ M^−1^/s^−1^) was previously reported, along with evidence of its rapid absorption in the stomach, with plasma concentrations elevated up to 220 µM [42]. Based upon these data, although 1000 mg of Vitamin C reduced extracellularly released O_2_^−^ generated by phagocytic NADPH oxidase, its effects diminished more quickly than the effect of 50 mg of PB-Blend. Importantly, unlike PB-Blend, Vitamin C’s potency was limited to NOX2 and did not show statistically significant inhibitory effects on the mitochondrial respiratory chain, or on iNOS-dependent ROS generation.

*Bioavailable NO*: Nitric oxide (NO), the main intracellular antiviral defense mediator, has been shown to inhibit a wide array of viruses, including COVID-19 [43]. Furthermore, NO can regulate cellular function, growth, and the death of immune cells, such as macrophages, neutrophils, T cells, and natural killer (NK) cells [44]. NO has a very short half-life, but is well stabilized and stored in blood cells when bound to hemoglobin (NOHb). Aside from the deleterious effects of acute ROS/NO dysregulation, it is known that chronic elevations in ROS are associated with the suppression of bioavailable NO and concomitant reductions in endothelial health. Consequently, it is reasonable to investigate the potential benefits of supporting impaired immune defense mechanisms after supplementation with natural, broad-spectrum-activity antioxidant products that may elevate bioavailable NO levels [32]. As mentioned earlier, PB-Blend was reported to increase the quantity of bioavailable NO in overweight and slightly obese persons [29]. In order to assess the effects on NO of a single dose of PB-Blend or Vitamin C, we analyzed the levels of hemoglobin-bound NO (NOHb), which has been previously described as a marker of endothelial function [45,46]. Our baseline results demonstrate low circulating NOHb concentrations in both study groups, which confirmed the development of endothelial dysfunction in response to excessive ROS caused by COVID infection.

**Figure 5:**
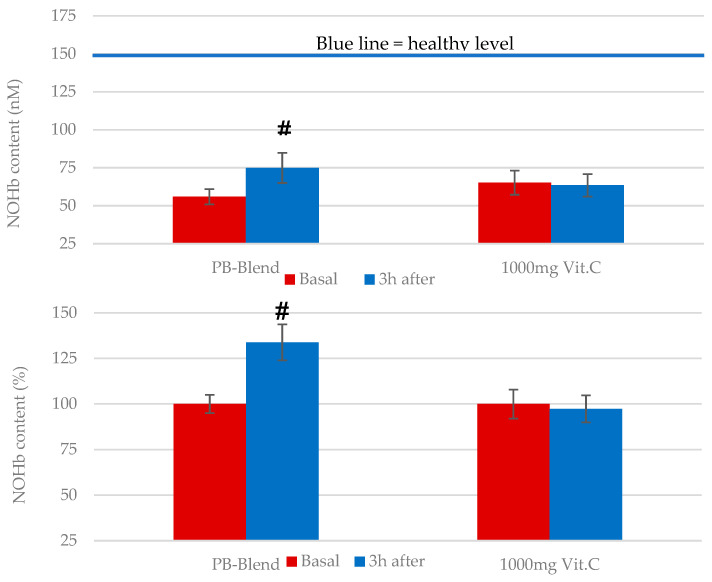
Single-dose effects of 50 mg of PB-Blend or 1000 mg of Vitamin C on time-dependent NOHb values in subjects 18–24 days after diagnosis of moderate course of COVID-19. The previous viral insult caused extreme elimination of bioavailable, circulating NO. Circulating NO prior to supplementation was up to 56.0 +/− 5 nM in PB-Blend group and up to 65.2 +/− 8 nM in Vit. C group, respectively. These values represent an almost three-times lower value compared to typical healthy NOHb levels (170–180 nM [32]) and illustrate that this study population was still in a recovery phase following infection. Data are shown as mean +/− standard error of the mean (SEM, *n* = 14 per group). Symbol #—*p* < 0.05, indicating statistical significance vs. value before supplementation.

The single encapsulated oral administration of 50 mg of PB-Blend resulted in a significant elevation in circulating NOHb levels after 3 h of ingestion (Figure 5). Vitamin C had no statistically significant effect on the NOHb levels under these experimental conditions.

*Immune (inflammatory) system response*: In clinical practice, acute inflammation is typically assessed by evaluating the increasing concentrations of leukocytes and neutrophils in cell lines, and by measuring the generic but generally accepted inflammatory markers such as hsCRP (highly sensitive C-reactive protein). For this study, we expanded the inflammatory analysis profile to include cytokines and interleukins such as TNFα, INFγ, IL1β, IL6, IL8, and IL10 in order to more comprehensively assess immune system responses to viral respiratory disease insult. All of these cytokines are generally accepted to play central roles in the activation of cellular immune defenses. Interestingly, however, our baseline results, presented in Table 2 below, revealed no elevations in the “conventional” inflammatory markers (hsCRP, leukocyte, or neutrophil count).

**Table 2:** Profiles of baseline inflammatory markers measured according to the study protocol and as described earlier in “Cytokine and interleukin plasma analysis”. Data represents mean value +/− standard error of the mean (SEM).

Treatment Group	Cov2-IgG	Cov2-IgM	IL1-ß	IL 6	IL 8	IL 10	TNF-α	IFN-γ	hsCRP	Leucocytes	Neutrophils
50 mg PB-Blend	275 ±142	2.5 ±1.6	0.1 ±0.02	0.8 ±0.1	5.7 ±1.1	0.1 ±0.02	4.5 ±0.3	0.4 ±0.1	0.4 ±0.1	5.2 ±0.2	2.8 ±0.2
1000 mg Vit.C	439 ±147	9.9 ±8.2	0.1 ±0.02	0.8 ±0.1	4.4 ±0.6	0.2 ±0.01	4.3 ±0.6	0.3 ±0.03	1.0 ±0.3	5.9 ±0.4	3.6 ±0.3
Ref. values	<33.8 BAU/mL	<10 BAU/mL	<5 pg/mL	<7 pg/mL	<62 pg/mL	<10.8 pg/mL	<8.1 pg/mL	<38.7 pg/mL	<1 mg/dL	3.9–10.4 10^3^/µL	1.9–7.3 10^3^/µL

It was of interest to us that our study groups exhibited baseline cytokine and interleukin profiles that were within the normal or low–normal ranges (similar to what we observed earlier with the conventional inflammatory markers) prior to supplementation. In contrast to what we observed here, many studies have described lymphocyte count changes (for example, changes in TNFα or IL6) as useful COVID-19 markers [39] and postulated that serum levels of IL6 and hsCRP show significant correlations with the severity of COVID-19 and can be used as independent factors in order to predict the risk of mechanical ventilation [47,48].

Our data did not support those earlier observations and instead showed low IL1β, IL6, IL8, and IL10 values at baseline and also after 3 h of PB-blend or Vitamin C supplementation. Admittedly, our population was no longer in the throes of “active infection” according to PCR testing. However, according to the testing presented here, this population was still, during their recovery phase, in a definite state of oxidative disbalance, and therefore could reasonably have been expected to manifest sustained elevations in these markers. Due to these unexpected observations, it appears that the use of conventional inflammatory markers, as well as interleukin profiles for the prediction or confirmation of complications related to viral insult, may not be entirely reliable, especially when compared to our data obtained using CMA. Further investigation and side-by-side comparisons are warranted in this context.

*Inflammatory resistance—TNFα*: Tumor necrosis factor alpha (TNFα) is an extremely versatile cytokine that has multiple effects on different cell types. It modulates release from corticotropin, the formation of acute-phase proteins such as hsCRP, the elicitation (but also limitation) of febrile response, the facilitation of the migration of inflammatory cells into tissue, the stimulation of phagocytosis, increased insulin resistance, and the activity of cyclooxygenase-2. TNFα is produced by many cells, including macrophages, lymphocytes, mast cells, endothelial cells, cardiomyocytes, fibroblasts, and neuronal tissue. In healthy organisms and at appropriate levels, it serves as a major regulator of inflammatory responses. However, it is also known to be involved in the pathogenesis of some inflammatory and autoimmune diseases [49].

Using the Inflammatory Resistance Assay (described earlier herein), we measured the relative abilities of PB-Blend and Vitamin C to reduce the plasma concentrations of TNFα-induced H_2_O_2_. Before and 3 h after treatment, our ex vivo assay tested the resistance of our volunteers’ blood cells exposed to 500-fold elevated levels of TNFα (40 ng/mL), comparable to the acute inflammation that would be expected during influenza or other severe viral infections. Then, following the same protocol, we repeated the ex vivo challenge, but this time we used a much higher TNFα concentration (200 ng/mL, comparable to the cytokine storm induced by COVID-19) [18].

**Figure 6:**
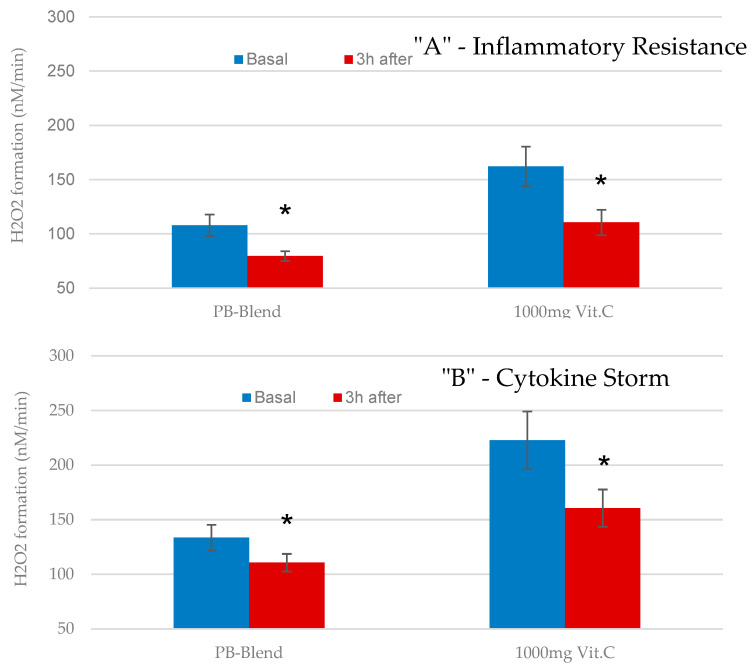
Single-dose effects of 50 mg of PB-Blend or 1000 mg of Vitamin C on time-dependent ex vivo TNFα-induced H_2_O_2_ formation rates in subjects with moderate course of COVID-19 infection. Panel (**A**): The TNFα level (40 ng/mL) is simulating the effects comparable to acute viral infection and described as “Inflammatory resistance”. Panel (**B**): Represents induction of H_2_O_2_ from blood cells after adding 200 ng/mL of TNFα, simulating a “Cytokine Storm” condition. Study volunteers received a single dose of PB-Blend or Vitamin C 18–24 days after diagnosis of COVID-19. Data are shown as mean +/− standard error of the mean (SEM, *n* = 14 per group). Symbol *—*p* < 0.01, indicating statistical significance vs. value before supplementation.

Under these experimental conditions, the inhibitory capacity of both tested materials was again similar (Figure 6B), with 50 mg of PB-Blend and 1000 mg of Vitamin C showing efficacy in modulating the cellular generation of H_2_O_2_ [31] Again, it should be noted that Vitamin C was administered at a 20-fold higher amount compared to PB-Blend.

## 3. Materials and Methods

**Figure 7:**
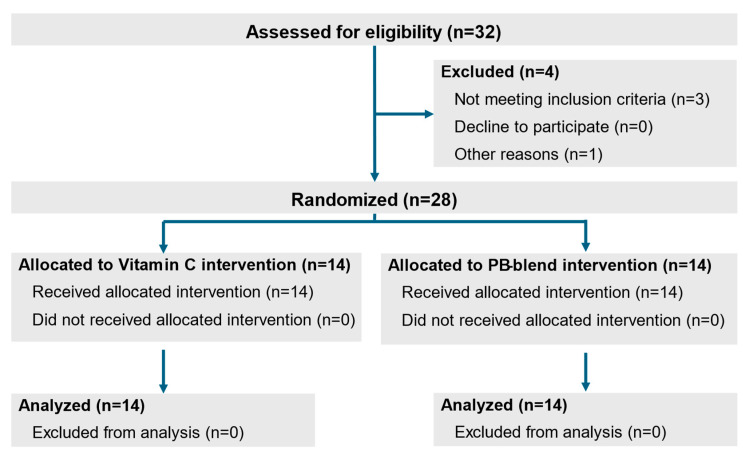
Study design flowchart.

*The Study design*: This study was designed and conducted under a randomized, double-blind, single-dose (acute) protocol. The study population consisted of 28 (13 males and 15 females) volunteers (see Figure 7) who had experienced a moderate COVID-19 disease course during the time period when the Alpha strain was the predominant Variant of Concern, and who also met the inclusion and exclusion criteria, as described below.

All subjects were enrolled 18–24 days after having tested positive for COVID-19 via PCR test. A “moderate COVID-19 disease course” was determined according to CDC criteria for a moderate disease course [50]. The assignment of volunteers into treatment groups was performed in a fully randomized manner. Neither the researchers nor the volunteers had any visibility or knowledge related to the group assignments or the respective identities of the test materials. After randomization, subjects were administered a single encapsulated dose of 50 mg of PB-Blend or a 1000 mg dose of Vitamin C as a positive control. Changes in the levels of bioavailable NO (measured as circulating NOHb) were assessed, as well as the ex vivo cellular formation of mitochondrial, NOX2-, iNOS-, and TNFα-dependent ROS. All parameters were measured in real time prior to ingestion (baseline), and then at 30, 60, 120, and 180 min after administration. All study volunteers were required to read and sign informed consent documentation. The study protocol was registered with and approved by the ethics committee of the Federal Medical Association of Baden-Württemberg (F-2021-033). The study was conducted according to the provisions of German law, ICH-GCP guidelines, and the general principles of the original World Medical Association’s Declaration of Helsinki.

*Definition of moderate COVID-19 infection*: Individuals experiencing moderate COVID-19 infection have any of the various signs and symptoms of COVID-19 (e.g., fever, cough, sore throat, malaise, headache, muscle pain, shortness of breath, dyspnea, or abnormal chest imaging) and show evidence of lower respiratory disease via clinical assessment or imaging and the saturation of oxygen (SpO_2_) ≥ 94% in room air at sea level.

*Inclusion criteria*:

- Confirmed COVID-19 infection;
- Negative COVID-19 rapid test on the day of examination;
- CDC criteria for return-to-work have been fulfilled;
- At least 10 days and up to 20 days have passed since symptoms first appeared;
- At least 24 h have passed since the last fever without the use of fever-reducing medications;
- Symptoms (e.g., cough or shortness of breath) have improved;
- Otherwise medically stable population;
- BMI between 24–30;
- Aged between 40 and 55 years.

*Exclusion criteria*: No intake of vitamins/supplements during the 2 weeks prior to inclusion was permitted. No medications known to affect endothelial function (e.g., β-blockers) were permitted. Subjects who had generally accepted contraindications to physical exercise, smokers, those with type 1 and type 2 diabetes, liver and kidney impairments, psychiatric disorders, other disorders of acute or chronic nature (gastrointestinal, pulmonary, renal, cardiac, neurological, or psychiatric disorders), known allergies to foods or their ingredients, those using weight-reducing preparations or appetite suppressants, and those having participated in a clinical study within the last 30 days prior to the beginning of this study or during this study were also excluded. Health status was checked via clinical and laboratory examination.

### 3.1. Material

The PB-Blend (VDF FutureCeuticals, Momence, IL, USA) is a patented, rationally developed blend of seven (7) plant-based extracts and powders, derived from blueberry, broccoli, cherry, green coffee bean, green tea, kale, and turmeric combined together in proprietary ratios and standardized to NLT 60% total polyphenols, 30% catechins, and 3% curcuminoids. Our testing indicates the following as a typical phytochemical profile for PB-Blend (Table 3). 

### 3.2. Methods

*Cellular Metabolic Activity (CMA) and Extended CMA (eCMA) Assay*: The cellular metabolic activity (CMA) and extended cellular metabolic activity (eCMA) analytical methodologies have been published [28,51,52]. These publications describe the participation of various ROS-generating metabolic pathways such as NADPH oxidase 1 (NOX1), mitochondria, and extracellular peroxidases in the generation of ROS as factors contributing to the collective CMA value. As a preliminary proof-of-concept exercise to further justify the current study, we conducted an earlier internal pilot study (unpublished, data available upon request) on a population having undergone a severe course of COVID-19. The results revealed that the CMA levels remained highly elevated after 147 (+/−16) days following a positive COVID-19 test. The data helped to justify our hypothesis that a COVID-19 study population could be suitable for our intended study.

**Figure 8:**
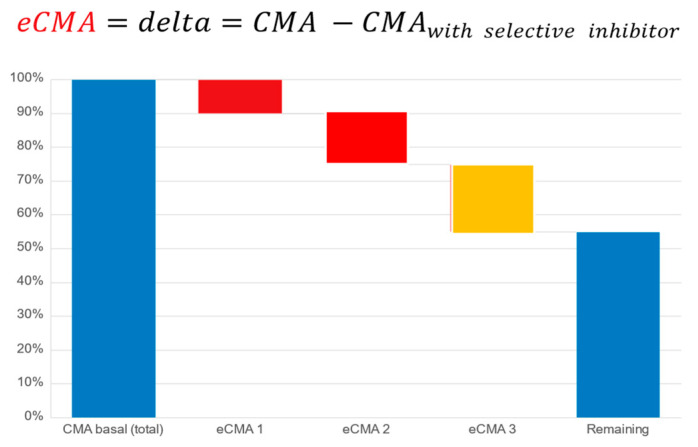
Principle of cellular metabolic activity and extended cellular metabolic activity assays designed to follow cellular physiology, health, and inflammatory marker changes. Illustrated schema represents an example distribution of oxidative stress-induced dysfunctional mitochondria (labeled as eCMA1), activated NOX2 of inflammatory cells (labeled as eCMA2), and dysfunctional (uncoupled) iNOS (labeled as eCMA3). Note that the total of the four bars on the right adds up to 100%.

The principle of the CMA assay is based upon the real-time monitoring of the cellular, mitochondrial, NADPH oxidase 1 or 2-, peroxidase-, and inducible nitric oxide synthase-dependent (iNOS-dependent) generation of reactive oxygen species. Cellular membrane and mitochondria-permeable spin probe 1-hydroxy-3-methoxycarbonyl-2.2.5.5-tetramethylpyrrolidine (CMH, 1 mM) dissolved in KHB buffer (20 mM; pH 7.4) was mixed with freshly drawn capillary blood to perform measurements of ROS generation under controlled temperature and oxygen concentrations (t = 36.6 °C, pO_2_ = 110 mm/Hg) [26]. For our extended CMA (eCMA) analysis, a portion of the sample was taken and kept in ice bath samples at 4 °C. It was mixed with the following: (a.) superoxide dismutase (SOD, 50 mU/mL; eCMA-ENDO) to measure amounts of extracellularly released O_2_^−^ by NOX1; (b.) catalase (50 mU/mL, eCMA-INFLA) to analyze peroxidase-dependent H_2_O_2_ formation; (c.) antimycin A (10 µM, eCMA-MITO) to evaluate mitochondria-dependent O_2_^−^ generation; (d.) Apocynin (10 µM, eCMA-PHAGO-NOX2) to detect phagocytic NADPH-dependent (NOX2-dependent) O_2_^−^ formation; and, (e.) 1400 W (0.1 µM, eCMA-iNOS) to identify inducible nitric oxide-dependent (iNOS-dependent) O_2_^−^/ONOO^−^ generation. The addition of oxygen label (NOX-15.1, 5 µM) to the blood sample made it possible for us to monitor the oxygen concentration of cellular, mitochondrial, NADPH oxidase-, and peroxidase-dependent oxygen consumption (see Figure 8). The EPR signal was detected using a Bruker EMXnano (Bruker Biospin GmbH, Ettlingen, Germany) equipped with a Noxygen Temperature and Gas controller (“NOXYSCAN System”, Noxygen, Elzach, Germany)). The EPR signal was simultaneously measured using a portable VitaScreen EPR spectrometer (Noxygen, Elzach, Germany) for internal equipment validation purposes. Calibration of the EPR signal was performed using a calibration solution with a standard concentration of CP° (500 µM), or oxygen label (NOX-15.1, 100 µM) filled into an oxygen-permeable 50 µL PTX capillary via the deoxygenation of oxygen label solution using the perfusion of pure nitrogen (99.99%).

**Figure 9:**
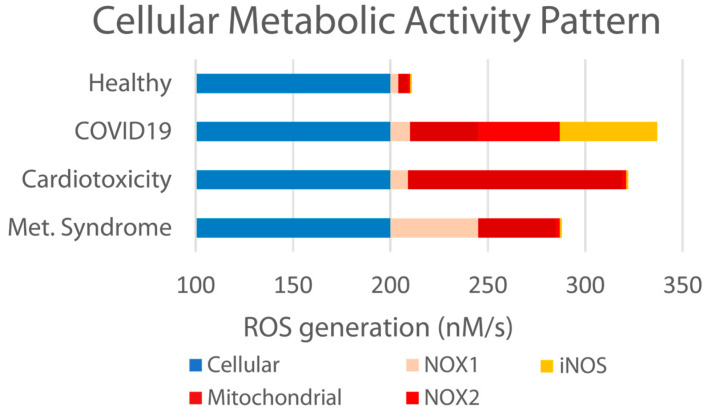
Effects of various health conditions on redox-dependent oxidative distress. According to each condition (healthy, viral insult, cardiotoxicity, and metabolic syndrome), enzymes such as NOX1, NOX2, iNOS, and enzymes of the mitochondria respiratory chain are involved in excessive but distinct generation of ROS, with each pattern representing the diagnostic “signature” of its respective pathology. Consequently, we examined the following various parameters to gain further insight into PB-Blend’s or Vitamin C’s potential capacities to support a healthier redox balance.

We and others [29,32,53,54,55] have previously reported that an “imbalanced” endothelial system results from various etiologies, including metabolic syndrome, cardiotoxicity, and other chronic conditions, as well as acute bacterial, viral, and environmental insult. Specifically related to viral insult, this is accompanied by elevated ROS formation, the diminished bioavailability of NO, and the elevation in other markers such as NOX1, NOX2, and iNOS (as illustrated in Figure 9 below).

*Inflammatory Resistance Assay*: This ex vivo assay describes changes in extracellular H_2_O_2_ generation by blood cells after (a.) exposure to tumor necrosis factor alpha (TNFα) at a final concentration of 40 ng/mL, representative of elevated TNFα plasma concentrations in human blood, and then (b.) exposure to 200 ng/mL (a 5-times-higher amount of TNFα and comparable to the amount of TNFα that could be observed in a person infected with COVID-19) in order to mimic the conditions of a cytokine storm [18]. The samples were mixed with a solution of human TNFα and spin probe 1-hydroxy-4-phosphono-oxy-2,2,6,6-tetramethyl-piperidine (PPH) dissolved in Krebs HEPES buffer (20 mM, pH 7.4) at the final concentrations of 40 ng/mL or 200 ng/mL TNFα and 1 mM PPH, respectively. After 60 min incubation in PTX oxygen-permeable capillaries, samples were placed in a capillary treatment chamber (NOX-E.7-CTC, Noxygen) at a temperature of 37 °C and 40 mmHg oxygen partial pressure. Samples were analyzed with the NOXYSCAN System and also partially in parallel with the VitaScreen EPR Spectrometer (Noxygen, Elzach, Germany) for internal equipment validation purposes.

*Bioavailable NO concentration assay*: Heparinized venous blood samples that were previously quick frozen in liquid nitrogen and stored at −80 °C were analyzed for NOHb content at −196 °C in a quartz finger Dewar. The EPR spectrometer was operating at 100 kHz field modulation to collect the EPR spectra at X-band 9.7 GHz using the following settings: microwave power: 50 mW; modulation amplitude: 8 G; center field: 2.01 g; sweep width: 60 G; conversion time: 20 ms; time constant: 80 ms; number of scans: 60; and total detection time: 600 s. The amount of detected NO^•^, a key signaling molecule of vascular physiology [45], was determined from the calibration curve for the intensity of the EPR signal of erythrocytes treated with known concentrations of nitrite (1–25 μM) and Na_2_S_2_O_4_ (20 mM).

*Cytokine and interleukin plasma analysis*: Cytokines and interleukins are conventionally accepted to have roles in immune responses during viral infection. Accordingly, we performed profiling of inflammatory markers such as IL1β, IL6, IL8, IL10, IFNγ, and TNFα. Inflammatory markers were measured using electrochemiluminescence-based V-Plex immunoassays (MesoScale Discovery, Gaithersburg, MD, USA) in plasma from samples collected before and after single-dose supplementation of PB-Blend or Vitamin C.

*Chemicals*: The spin probes 1-hydroxy-3-methoxycarbonyl-2.2.5.5-tetramethylpyrrolidine (CMH, NOX-02.5-VIT), 1-hydroxy-4-phosphono-oxy-2.2.6, and 6-tetramethylpiperidine (PPH, NOX-03.2); EPR-Krebs HEPES buffer VIT (KHB-VIT, NOX-21.2-VIT) containing metal chelators deferoxamine (DF, NOX-09.1) and diethyldithiocarbamate (DETC, NOX-10.1) and Heparin (100 U/mL) and Krebs HEPES buffer (KHB, NOX-07.6); oxygen label (NOX-15.1); and the eCMA working solutions eCMA MITO (NOX-22.1-VIT), eCMA ENDO (NOX-23.1-VIT), eCMA-INFLA (NOX-24.1-VIT), eCMA-iNOS (NOX-26.1-VIT), and eCMA PHAGO NOX2 (NOX-25.1-VIT) were obtained from Noxygen Science Transfer and Diagnostics (Elzach, Germany). All other chemicals and reagents used were of analytical grade and purchased from Sigma-Aldrich (St. Louis, MO, USA) unless otherwise specified.

### 3.3. Statistical Analysis

Prior to performing statistical analyses, descriptive statistics were determined for each dependent variable, and normality was assessed. The sample size was determined via power analysis using XLSTAT (2020.4.1, Build 1018) to reach a statistical significance of *p* < 0.01 for CMA and NOHb and a power of 0.8. The effect strength for these parameters was derived from the earlier reported single-dose effect data. The outliers were determined using a Tukey fences approach (Q_1_ − 1.5 × (IQR) and Q_3_ + 1.5 × (IQR), where Q_1_ is the first quartile, and Q_3_ is the third quartile). Outliers exceeding these boundaries were excluded from the analysis. Repeated measures ANOVAs were performed with the group as the between-subject factors. In the case of interactions, univariate ANOVAs were performed to determine the nature of the interaction to establish appropriate inferences. The results are presented as the mean ± standard error of the mean (SEM). Statistical significance was determined at the *p*-level of <0.05 or <0.01.

## 4. Conclusions

This is the first study showing real-time elevations in the iNOS and NOX2 enzymatic systems in a population recovering from a moderate course of COVID-19. We were able to quantify the mitochondrial activity that can lead to uncontrolled ROS generation and subsequent damage of the endothelial system. Due to the great number of similarities in terms of the immune disbalance engendered by all respiratory viruses, we believe that these data provide a reasonable explanation for some of the numerous complications so often observed in persons suffering from respiratory viral infection.

Altogether, these results suggest that 50 mg of PB-Blend, a plant-based dietary supplement that exhibits a broad spectrum of antioxidant activities, has potential to modulate the therapeutic targets that can become disbalanced due to viral infection. As evidenced during our CMA/eCMA assessments, orally administered PB-Blend inhibited iNOS, NOX2, and mitochondria-dependent ROS generation. Comparatively, according to these data, 1000 mg of Vitamin C (at a 20-fold higher dosage than the PB-Blend) revealed a more narrowly focused antioxidative activity, limited to the inhibition of the NOX2-dependent generation of ROS.

As previously discussed, the particular patterns of the ROS elevations associated with respiratory viral insult are not identical to those associated with other illnesses or conditions such as heart disease or diabetes. Any exogenous therapeutic support requires a robust combination of antioxidative activities in order to best regulate the many specific targets that together drive disbalance and the associated uncomfortable, debilitating, and at their worst dangerous symptoms observed during the course of viral infection. PB-Blend’s observed ability to increase the bioavailable HbNO and modulate the enzymatic systems related to iNOS, NOX2, and mitochondria indicates such broad-spectrum antioxidative activity.

This study has several obvious limitations. Recruitment was difficult during the pandemic, and the identification and timely enrollment of candidates recovering from COVID-19 that met the inclusion criteria was a challenge. Consequently, our N was not as large as we might have preferred. The statistical import could have been enhanced by a larger study population. This study had a positive control, but lacked a placebo. Also, this study only examined acute, single-dose effects. Because this was the first study of its kind on PB-Blend, there are opportunities to expand our understanding through the refinement and alteration of future study designs. Placebo-controlled, higher-N studies should examine the impact of both the acute and longitudinal supplementation of PB-Blend compared to a placebo, and potentially other natural antioxidants, including Vitamin C, on dysregulated redox signaling. A cross-over design could also be implemented. Healthy and virally infected populations could be combined in order to further compare and control PB-Blend’s effects on the observed pathways. Studies could also include subjective assessments (VAS or QOL, etc.) to better quantify the changes in the severity of study participants’ perceived symptoms during and after treatment.

However, to the best of our knowledge, PB-Blend is the only natural dietary supplement that has been shown to exhibit all of the reported activities. In addition to providing daily baseline support against excessive ROS production in generally healthy populations, PB-Blend could be considered a novel alternative to, or to be used in tandem with Vitamin C for applications to promote immune health, recovery, and a potential reduction in associated feelings of illness and malaise during times of heightened respiratory viral risk. Finally, and as illustrated herein, the degree and composition of these eCMA pattern analyses may provide clinicians and researchers with a novel tool to assess the effects of acute and chronic forms of immune disbalance. Direct assessments of the various NOX pathways could be developed as hallmarks of damaged immune and cardiovascular systems.

## Figures and Tables

**Table 1:** Baseline clinical and laboratory parameters according to inclusion and exclusion criteria described above in study protocol. Data represent mean values +/− standard error of the mean and corresponding reference values. The presented values are as follows: sBP—systolic blood pressure, dBP—diastolic blood pressure, HR—heart rate, FMD—flow-mediated dilatation, pO_2_—oxygen partial pressure, FVC—forced vital capacity, HbA1C—% of glycosylated hemoglobin. The basic laboratory parameters were analyzed in the medical care center at Clotten (MVZ Clotten, Freiburg, Germany) with an established comprehensive QM system. Data are shown as mean +/− standard error of the mean (SEM).

Study Groups	sBP	dBP	HR	FMD	pO_2_	FVC	Glucose (Fasting)	Creatine	HbA1C	Total-Chol.	LDL-Chol.
50 mg PB-Blend	119 ±3	77 ±2	66 ±4	9.3 ±0.6	97 ±0.5	4.1 ±0.3	114 ±9	0.7 ±0.03	5.4 ±0.1	190 ±10	126 ±10
1000 mg Vit.C	126 ±4	76 ±4	64 ±2	9.9 ±0.7	98 ±0.3	3.6 ±0.2	114 ±9	0.7 ±0.04	5.4 ±0.2	198 ±8	133 ±9
Ref. values	≤130 mmHg	≤90 mmHg	≤70 1/min	≥10 %	>94 %	4-5 L	≤100 mg/dL	0.5–1 mg/dL	<7 %	<200 mg/dL	<116 mg/dL

**Table 3:** Phytochemical composition of PB-Blend in 50 mg serving dose.

Phytochemical Components	Units	50 mg	Amount (%)
Catechins	mg	18.4	36.8
Chlorogenic acids	mg	13.2	26.2
EGCG	mg	9.2	18.4
Curcumin	mg	2.1	4.2
Caffeine	mg	1.7	3.4
Anthocyanins	µg	52.5	0.001
Trigonelline	µg	55.1	0.001
Vitamin C	ng	5.2	1 × 10^−4^
Vitamin E	ng	0.15	3 × 10^−6^

## Data Availability

The data presented in this study are available on request from the corresponding author. Data are not publically available due to privacy restrictions associated with personalhealth data of volunteers.
